# Research progress on de-escalation strategies of dual antiplatelet therapy after acute coronary syndrome

**DOI:** 10.3389/fmed.2026.1797759

**Published:** 2026-07-28

**Authors:** Xinyu Liu, Jingjing Liu, Chang Xu

**Affiliations:** Cardiovascular Ward, Department of Geratology, The First Hospital of China Medical University, Shenyang, China

**Keywords:** acute coronary syndrome, Chinese populations, dual antiplatelet therapy, percutaneous coronary intervention, STEMI

## Abstract

**Introduction:**

Dual antiplatelet therapy (DAPT) is standard after percutaneous coronary intervention (PCI) in acute coronary syndrome (ACS), but prolonged DAPT increases bleeding risk, particularly in East Asian and high bleeding-risk (HBR) populations. De‑escalation strategies aim to balance ischaemic and bleeding risks.

**Methods:**

We conducted a structured literature search in PubMed, EMBASE, and the Cochrane Library (January 2018–April 2026) for randomized trials and key studies on DAPT de-escalation in ACS patients after PCI, using the Academic Research Consortium (ARC) tripartite classification framework.

**Results:**

Strong evidence supports discontinuation-based strategies: 3-month (TICO, TWILIGHT, SMART-CHOICE) and 1-month (ULTIMATE-DAPT, T-PASS, TARGET-FIRST) DAPT followed by P2Y12 inhibitor monotherapy reduce bleeding without increasing ischaemic events in selected patients. MASTER-DAPT established 1-month DAPT safety in HBR patients. However, very early aspirin withdrawal (within 4 days, NEO-MINDSET) failed non-inferiority for ischaemic outcomes, and clopidogrel monotherapy after 1-month DAPT was associated with higher ischaemic risk (STOPDAPT-2 ACS). Drug‑intensity de-escalation (e.g., ticagrelor-to-clopidogrel switching) and guided strategies using genotyping or platelet‑function testing show promise but require further validation.

**Conclusion:**

Individualized DAPT de-escalation, guided by dynamic risk assessment using PRECISE‑DAPT and ARC-HBR criteria, optimizes net clinical benefit. Ticagrelor monotherapy after at least 1 month of DAPT is preferred over clopidogrel in ACS. Future research should focus on precision antithrombotic strategies in Chinese populations and high‑risk subgroups.

## 1 Introduction

Acute coronary syndrome (ACS) remains a leading cause of death and disability in China, with its pathophysiology primarily driven by rupture or erosion of atherosclerotic plaques and subsequent thrombus formation, resulting in acute myocardial ischemia or necrosis (1, 2). Timely restoration of culprit vessel blood flow is critical for improving prognosis, and percutaneous coronary intervention (PCI) has become the mainstay for revascularization in ACS (3).

Since the early 2000s, dual antiplatelet therapy (DAPT)—typically aspirin combined with a P2Y ₁₂ receptor inhibitor—has been established as the cornerstone of secondary prevention after PCI in ACS patients (4). Multiple randomized trials have demonstrated that standard DAPT regimens, usually administered for 12 months, effectively reduce the risk of stent thrombosis, myocardial infarction, and other major adverse cardiac and cerebrovascular events (MACCE), leading to guideline recommendations worldwide (5).

However, with the advent of second- and new-generation drug-eluting stents, refinement of interventional techniques (such as intravascular ultrasound- or optical coherence tomography-guided PCI), and improvements in perioperative management, the incidence of post-PCI ischemic events has significantly declined (6). In contrast, prolonged and potent DAPT increases bleeding risk, particularly in elderly patients, those with low body weight, renal impairment, or in East Asian populations. Studies have shown that bleeding events not only elevate hospitalization and mortality risks but may also negate the long-term benefits of ischemic protection (7).

Notably, a growing body of clinical evidence has indicated that East Asian patients have a paradoxically higher risk of serious bleeding during antiplatelet therapy compared with Western populations, coupled with a relatively lower risk for cardiovascular events—a phenomenon termed the “East Asian paradox” (8). The 2025 updated position statement from JACC: Asia describes that East Asian patients experience reduced anti-ischemic benefits and increased bleeding risk during antithrombotic therapies compared with Caucasian patients (9). This unique risk–benefit trade-off may be influenced by a complex interplay of genetic and environmental factors, including specific atherothrombotic cardiovascular risks, *Helicobacter pylori* infection, sites of cranial atherosclerosis, and low body weight (9). For example, an updated multiethnic analysis demonstrated that Chinese patients had persistently elevated bleeding odds with ticagrelor compared with clopidogrel, whereas Indian patients showed a lower bleeding risk, arguing against treating “Asian” populations as a single category in net clinical benefit calculations (10). This highlights the need to optimize DAPT strategies in East Asian populations based on their unique risk–benefit trade-off, rather than directly extrapolating evidence from Western cohorts (11).

From a pathophysiological perspective, ACS presents a temporal risk profile: the early phase, particularly the first 1–3 months post-PCI, is marked by heightened thrombotic risk. Over time, as plaques stabilize, endothelial repair progresses, and inflammation subsides, ischemic risk gradually decreases, whereas bleeding risk persists or relatively increases (12). Against this backdrop, DAPT de-escalation—adjusting antiplatelet intensity according to the dynamic balance of ischemic and bleeding risk—has gained attention as a strategy to maximize net clinical benefit.

**Figure 1:**
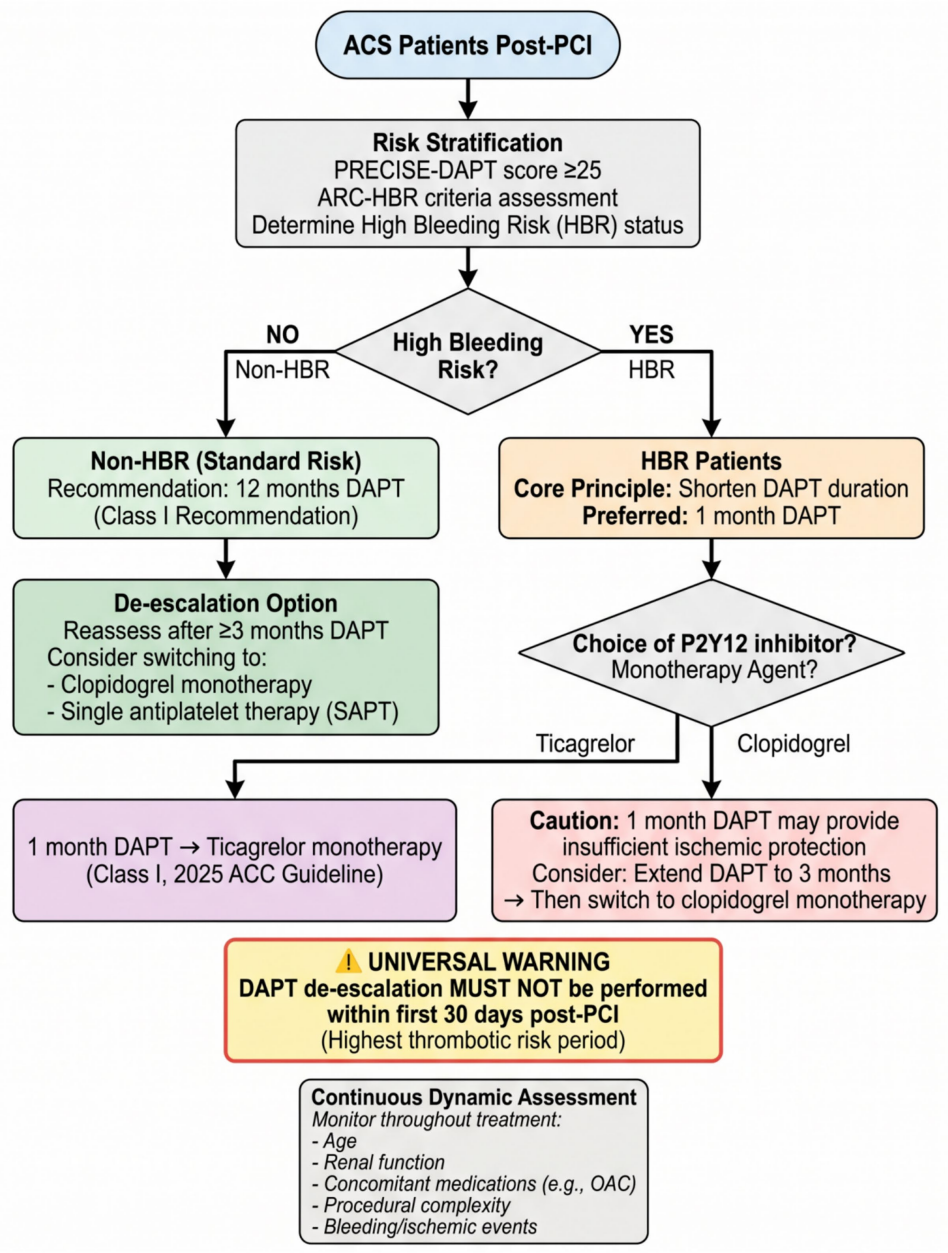
Clinical decision pathway for DAPT De-escalation in ACS patients after PCI. The process begins with risk stratification using PRECISE-DAPT score (≥25) and ARC-HBR criteria. For non-HBR patients, 12 months of DAPT is the default (Class I). For HBR patients, shortened DAPT (1 month) is recommended, but the choice of monotherapy agent matters: ticagrelor monotherapy after 1 month is safe (Class I), whereas clopidogrel monotherapy after 1 month may provide insufficient ischemic protection, warranting longer DAPT (e.g., 3 months) before de-escalation. De-escalation should never be performed within the first 30 days post-PCI. Decision-making integrates dynamic reassessment of patient-specific factors.

According to the 2023 Academic Research Consortium (ARC) consensus document (13), de-escalation can be achieved through three distinct approaches: (1) discontinuation of one antiplatelet agent; (2) switching from a potent P2Y12 inhibitor to a less potent agent (e.g., clopidogrel); and (3) dose reduction of antiplatelet agents (11). This review adopts this tripartite classification framework to systematically synthesize current evidence. Recent randomized trials have explored the safety and efficacy of these strategies in specific ACS populations, though their optimal application, timing, and long-term benefits remain under debate (3, 14). This review summarizes the recent evidence on DAPT de-escalation after PCI in ACS patients, emphasizing the clinical outcomes, target populations, and potential risks of different approaches. We also discuss individualized antiplatelet therapy strategies in East Asian populations in light of the latest guideline recommendations, aiming to provide evidence-based guidance for clinical practice. The overall structure and key decision pathways are illustrated in Figure 1.

## 2 De-escalation by switching to less potent antiplatelet agents (drug-intensity de-escalation)

Drug-intensity de-escalation refers to reducing the potency of antiplatelet therapy within the framework of DAPT, primarily through switching from a potent P2Y12 inhibitor (ticagrelor or prasugrel) to clopidogrel, or through dose adjustment of antiplatelet agents. The goal is to maintain sufficient antithrombotic efficacy while minimizing bleeding risk. Within this domain, strategies can be further classified into unguided approaches (switch without testing) and guided approaches (switch guided by genetic or functional testing).

### 2.1 Alternative cyclooxygenase (COX) inhibitors

Aspirin, a non-selective COX inhibitor, irreversibly suppresses COX-1 to reduce thromboxane A₂ production, but concurrently inhibits endothelial COX-2, decreasing prostacyclin synthesis and increasing gastrointestinal bleeding risk (15). Indobufen, a selective and reversible COX-1 inhibitor, theoretically retains antiplatelet efficacy while mitigating gastrointestinal toxicity (16).

Clinical trials in stable coronary artery disease or aspirin-intolerant patients, such as OPTION (17) and ASPIRATION (18), demonstrated that indobufen combined with a P2Y ₁₂ inhibitor significantly reduced BARC type ≥2 bleeding events, without increasing major adverse cardiac and cerebrovascular events (MACCE) compared with aspirin. However, in ACS subgroups, indobufen was associated with higher ischemic risk, likely due to weaker platelet inhibition (19). Therefore, this strategy is currently limited to aspirin-intolerant patients or those at high gastrointestinal bleeding risk and is not recommended as routine therapy for ACS.

### 2.2 Switching from potent P2Y₁₂ inhibitors to clopidogrel

Potent P2Y₁₂ inhibitors, such as ticagrelor and prasugrel, have demonstrated superior efficacy over clopidogrel in ACS patients (20). In the PLATO trial, ticagrelor reduced 12-month MACCE risk by 16% compared with clopidogrel (HR 0.84, 95% CI 0.77–0.92) (21). However, the associated increase in bleeding risk has prompted exploration of “potent-to-mild” de-escalation strategies.

The TOPIC trial first showed that inACS patients who remained event-free 1 month after PCI, switching from ticagrelor or prasugrel to clopidogrel reduced 1-year net adverse clinical events (NACE) by 52% (*p* < 0.01), mainly due to reduced bleeding (22). TALOS-AMI further validated this strategy in 2,697 myocardial infarction patients: after 1 month of ticagrelor plus aspirin, switching to clopidogrel plus aspirin significantly lowered major bleeding at 12 months (2.3% vs. 3.4%, *p* < 0.001) without increasing MACCE (4.6% vs. 5.7%, *p* = 0.16) (23).

Timing is critical. A domestic retrospective study indicated that switching to clopidogrel within 30 days of ACS onset increased ischemic events by 93%, underscoring the risk of early de-escalation during the high-thrombotic phase (24). Accordingly, the 2025 American College of Cardiology (ACC) guidelines classify this approach as a Class IIb recommendation (Level B evidence), suggesting it only for high bleeding-risk (HBR) patients and not earlier than 1 month post-PCI (25).

### 2.3 Individualized (guided) de-escalation using genetic or functional testing

Precision de-escalation strategies utilize CYP2C19 genotyping or platelet function testing to guide P2Y ₁₂ inhibitor selection, representing a guided approach distinct from unguided switching (26). The TROPICAL-ACS trial employed platelet function testing to guide prasugrel-to-clopidogrel switching, showing a trend toward reduced bleeding without increased ischemic events (27). In STEMI patients, the POPULAR GENETICS trial used CYP2C19 genotype-guided therapy, resulting in significantly lower major bleeding in the genotype-guided group (9.8% vs. 12.5%, *p* = 0.04), with no difference in ischemic endpoints (28).

However, the TAILOR-PCI study, which included 5,302 PCI patients (82% ACS), found no overall superiority of genotype-guided de-escalation over standard therapy (29). A 2024 multicenter real-world study showed that genetic guidance significantly reduced BARC 2–5 bleeding, but benefits were primarily observed in HBR patients. While promising, individualized strategies require further high-quality randomized trials to confirm broad clinical net benefit (30).

### 2.4 Dose reduction of P2Y₁₂ inhibitors

Dose reduction of potent P2Y₁₂ inhibitors, without changing the drug, represents another de-escalation pathway. In PEGASUS-TIMI 54, long-term ticagrelor 60 mg twice daily in prior MI patients demonstrated non-inferior efficacy for the 3-year composite of cardiovascular death, MI, or stroke compared with 90 mg twice daily, with lower discontinuation due to adverse events (31).

This strategy is particularly relevant for East Asian populations. The Japanese PRASFIT-ACS trial showed that low-dose prasugrel (3.75 mg/day) in ACS patients achieved efficacy comparable to standard-dose clopidogrel (32). Chinese pharmacodynamic studies further indicate that ticagrelor 45 mg twice daily or 90 mg once daily produces stronger platelet inhibition than clopidogrel, potentially with lower bleeding risk (33). A single-center prospective study in STEMI patients comparing ticagrelor 60 mg vs. 90 mg twice daily showed no significant difference in 6-month MACCE or major bleeding. Nonetheless, large-scale outcome trials are lacking, and current guidelines do not formally recommend dose-reduction strategies (34).

**Figure 2:**
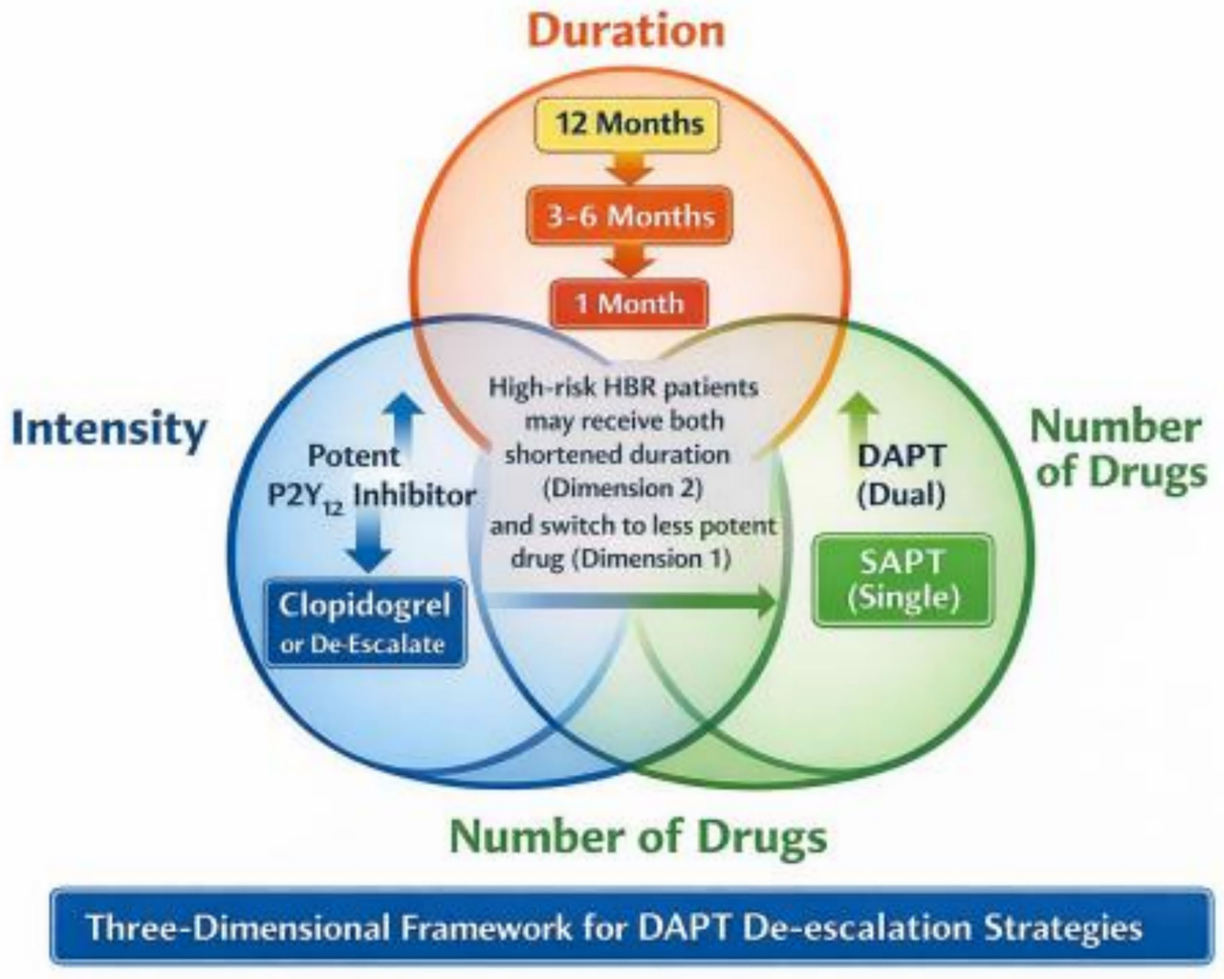
Three-dimensional conceptual framework of DAPT de-escalation strategies. This framework illustrates the three core dimensions of DAPT de-escalation and their synergistic relationships. The blue dimension (Intensity) represents switching from a potent P2Y ₁₂ inhibitor (e.g., ticagrelor) to clopidogrel. The orange dimension (Duration) refers to shortening DAPT from the standard 12 months (e.g., to 3–6 months or 1 month). The green dimension (Drug Number) represents transitioning from dual antiplatelet therapy (DAPT) to single antiplatelet therapy (SAPT). The intersection of the three rings indicates that, in high bleeding-risk (HBR) patients, multiple de- escalation strategies can be combined (e.g., shortening duration while switching drugs) to achieve individualized risk balancing.

In summary, drug-intensity de-escalation offers multiple options, including switching drug types, reducing doses, or shortening DAPT duration—the latter representing another core dimension of de-escalation (Figure 2).

## 3 De-escalation by discontinuation (DAPT duration shortening and ASA-free strategies)

The most compelling evidence for DAPT de-escalation comes from discontinuation-based strategies. According to the ARC classification, this involves discontinuing one antiplatelet agent after a period of DAPT, transitioning to single antiplatelet therapy (SAPT) with either a P2Y12 inhibitor or aspirin. The advent of new-generation drug-eluting stents (DES) has substantially reduced the risk of late stent thrombosis after PCI, providing a rationale for shortening DAPT duration. Successful implementation requires accurate identification of true HBR patients and applying de-escalation during periods of relatively lower ischemic risk.

### 3.1 Shortened DAPT to 3–6 months followed by P2Y12 inhibitor monotherapy

A substantial body of evidence supports 3-month DAPT followed by P2Y12 inhibitor monotherapy. The TWILIGHT trial enrolled 7,119 patients at high ischemic or bleeding risk, randomizing them after 3 months of DAPT to ticagrelor monotherapy vs. continued DAPT. Ticagrelor monotherapy reduced BARC 2–5 bleeding by 44% (*p* < 0.001) without increasing ischemic events (35). This benefit was consistent in NSTEMI and HBR subgroups (36). Similarly, the TICO trial, which included 3,056 ACS patients, demonstrated that switching to ticagrelor monotherapy after 3 months of DAPT significantly reduced 1-year NACE compared with 12-month DAPT (4.1% vs. 5.9%, *p* = 0.03), primarily due to reduced major bleeding (37). The SMART-CHOICE ACS subgroup analysis further supported the 3-month DAPT strategy followed by clopidogrel monotherapy, showing non-inferiority for MACCE with significantly lower bleeding. These 3-month strategies provide a balanced approach for ACS patients not at extremely high ischemic risk.

### 3.2 Shortened DAPT to 1 month: evidence and controversies

The MASTER-DAPT trial demonstrated that 1-month DAPT was non-inferior to longer DAPT durations (≥3 months) in high bleeding-risk (HBR) patients, with significant bleeding reduction (38). Its prespecified subgroup analysis focusing on patients presenting with ACS (acute or recent myocardial infarction) confirmed that 1-month DAPT was also safe and effective in HBR-ACS patients: the risk of cardiovascular events did not differ between 1-month and longer DAPT, while 1-month DAPT significantly reduced BARC type 2–5 bleeding (38). These findings support the feasibility of a 1-month DAPT strategy in HBR patients presenting with ACS, regardless of stent type or the specific single antiplatelet therapy (SAPT) used afterwards (38).

However, the dedicated STOPDAPT-2 ACS trial raised important concerns. In this pooled analysis of 4,136 ACS patients (derived from the STOPDAPT-2 trial and the STOPDAPT-2 ACS cohort), 1-month DAPT followed by clopidogrel monotherapy failed to achieve non-inferiority for the primary composite endpoint of cardiovascular and bleeding outcomes compared with standard 12-month DAPT. The 1-year cumulative incidence of the primary outcome was 3.20% with clopidogrel monotherapy vs. 2.83% with 12-month DAPT, and there was a trend toward more cardiovascular events (2.76% vs. 1.86%) despite a reduction in major bleeding (0.54% vs. 1.17%) (39, 40). These findings indicate that clopidogrel monotherapy after only 1 month of DAPT may not provide adequate ischemic protection in unselected ACS patients, even though the same strategy proved non-inferior in a mixed population of stable coronary artery disease and ACS patients (the original STOPDAPT-2 trial).

The STOPDAPT-3 trial evaluated prasugrel monotherapy (3.75 mg/day) started immediately after PCI in ACS or HBR patients and reported a significantly higher incidence of stent thrombosis (0.58% vs. 0.17%, HR 3.04) (41). In contrast, the ULTIMATE-DAPT study showed that switching to ticagrelor monotherapy after 1 month of DAPT (aspirin + ticagrelor) reduced bleeding (1.3% vs. 2.5%, *p* = 0.03) while maintaining comparable MACCE rates (4.1% vs. 4.8%, *p* = 0.62) (5). The T-PASS study transitioned patients to ticagrelor monotherapy after a median of 16 days post-discharge without increased ischemic risk (42).

Collectively, these seemingly discrepant results highlight a critical distinction: the choice of P2Y₁₂ inhibitor for monotherapy matters substantially in the ACS setting. While both ticagrelor and clopidogrel monotherapy significantly reduce bleeding to a similar extent, ticagrelor – but not clopidogrel—is associated with reduced ischemic outcomes compared with standard DAPT. This differential effect is largely driven by the STOPDAPT-2 ACS trial, in which clopidogrel monotherapy after 1–2 months of DAPT was linked to an increased risk of cardiovascular events (39, 40). The differences in ischemic protection are particularly evident when DAPT is discontinued between 1 and 3 months, whereas the increased ischemic harm associated with clopidogrel monotherapy may be mitigated when DAPT is continued for more than 3 months. Therefore, when aspirin withdrawal is planned after a shortened course of DAPT, ticagrelor should be preferred over clopidogrel (unless contraindicated) to maintain adequate ischemic protection in ACS patients.

Very recent data from ESC Congress 2025 have refined our understanding of ultra-short DAPT. The NEO-MINDSET trial (3,410 ACS patients) tested potent P2Y₁₂ inhibitor monotherapy initiated within 4 days of hospitalization vs. standard 12-month DAPT. The primary ischemic composite endpoint occurred in 119 patients in the monotherapy group vs. 93 in the DAPT group (*p* = 0.11 for non-inferiority), and stent thrombosis occurred in 12 patients vs. 4, respectively (43). Landmark analysis suggested that excess ischemic risk with monotherapy occurred in the first 30 days, with comparable outcomes thereafter. A prespecified NEO-MINDSET substudy further revealed differential effects by ACS subtype: among STEMI patients, early aspirin withdrawal was associated with higher ischemic events at 1 year (8.2% vs. 5.2%, HR 1.60, 95% CI 1.14–2.24), whereas in NSTE-ACS patients, ischemic event rates were comparable between monotherapy and DAPT (5.1% vs. 6.0%, HR 0.84, 95% CI 0.39–2.02, P for interaction = 0.030) (43). Bleeding was reduced with monotherapy in both subgroups. These findings indicate that very early aspirin withdrawal is not universally safe, particularly in STEMI.

By contrast, the TARGET-FIRST trial (published simultaneously in NEJM) demonstrated that in low-risk acute MI patients who had achieved early complete revascularization and completed 1 month of DAPT without complications, P2Y₁₂ inhibitor monotherapy was non-inferior to continued DAPT for adverse cardiovascular events (2.1% vs. 2.2%, *p* = 0.02 for non-inferiority) and resulted in lower major bleeding (2.6% vs. 5.6%, *p* = 0.002 for superiority) (44). This suggests that after an initial 1-month DAPT stabilization period, aspirin withdrawal can be safe in selected low-risk patients.

Taken together, the 2025 ACC/AHA guidelines state that patients at high bleeding risk can transition to SAPT (either aspirin or a P2Y₁₂ inhibitor) after 1 month (Class IIb recommendation), and de-escalation from ticagrelor or prasugrel to clopidogrel may be reasonable at least 1 month after PCI (Class IIb recommendation). The guidelines further recommend ticagrelor monotherapy starting 1 month post-PCI in ACS patients at high bleeding risk as a Class I recommendation (Level A evidence) (45).

### 3.3 Avoiding DAPT interruption within 30 days post-PCI

Studies including HORIZONS-AMI consistently demonstrate that the first 30 days after PCI represent a high thrombotic-risk period in ACS patients (46). The 2023 ESC guidelines explicitly recommend against interrupting DAPT during this period (Class III, Level B evidence) (47). Therefore, any de-escalation strategy should ensure at least 1 month of standard DAPT as a prerequisite. The NEO-MINDSET findings strongly reinforce this principle, as the excess ischemic risk with immediate aspirin withdrawal was concentrated in the first 30 days post-PCI (48).

## 4 De-escalation by discontinuation: high bleeding-risk populations and ASA-free strategies

### 4.1 High bleeding-risk populations: the MASTER-DAPT evidence

Patients at high bleeding risk (HBR) represent a distinct population in whom de-escalation strategies have been most extensively validated. The ARC-HBR criteria provide a standardized definition, and the PRECISE-DAPT score (threshold ≥25) offers a quantitative tool for risk stratification (49).

The MASTER-DAPT trial enrolled patients meeting HBR criteria and demonstrated that 1-month DAPT was non-inferior to longer DAPT durations for net adverse clinical events, with significant bleeding reduction (50). Importantly, the prespecified subgroup analysis of MASTER-DAPT focusing on patients with acute or recent myocardial infarction (i.e., ACS) confirmed the safety and efficacy of 1-month DAPT in ACS patients at high bleeding risk, establishing this as a guideline-supported strategy (51, 52).

When applying these risk assessment tools, clinicians should note that PRECISE-DAPT (score ≥25) and ARC-HBR criteria show moderate concordance. A recent analysis indicated that ARC-HBR identifies approximately 27% of post-PCI patients as high risk, whereas PRECISE-DAPT classifies 38% as high risk, with ARC-HBR demonstrating higher sensitivity but lower specificity for bleeding prediction (53). In clinical decision-making for DAPT de-escalation, the presence of any major ARC-HBR criterion (e.g., severe chronic kidney disease, gastrointestinal bleeding, oral anticoagulation requirement) or a PRECISE-DAPT score ≥25 should prompt consideration of shortened DAPT regimens, provided ischemic risk is not excessively high (54).

### 4.2 ASA-free strategies: STOPDAPT-3 and emerging evidence

The STOPDAPT-3 trial tested prasugrel monotherapy immediately after PCI in ACS or HBR patients and reported a higher incidence of stent thrombosis (0.58% vs. 0.17%, HR 3.04), cautioning against routine immediate aspirin withdrawal (55). This aligns with the NEO-MINDSET finding that very early aspirin withdrawal (within days) is not safe in unselected ACS patients (56).

However, the TARGET-FIRST trial demonstrated that after 1 month of uneventful DAPT, aspirin withdrawal with continuation of a P2Y12 inhibitor is safe and reduces bleeding in low-risk acute MI patients who have undergone complete revascularization (57). The upcoming LEGACY trial is expected to provide further evidence on the optimal timing and patient selection for aspirin withdrawal. Until then, current evidence supports maintaining at least 1 month of DAPT in ACS patients, with aspirin withdrawal considered thereafter in selected low-risk or HBR patients but not universally recommended across all ACS populations.

## 5 De-escalation by discontinuation: single-agent maintenance after DAPT

Traditionally, guidelines recommend lifelong aspirin after DAPT. Recent studies, however, have challenged this paradigm.

The HOST-EXAM study, although conducted in chronic coronary syndrome (CCS) rather than ACS patients, provided important insights. Its 5-year follow-up revealed that after 6–12 months of DAPT, clopidogrel monotherapy significantly reduced composite outcomes—including death, MI, stroke, repeat revascularization, and BARC 2–5 bleeding—compared with aspirin (HR 0.78, 95% CI 0.67–0.91). Current 10-year follow-up data have confirmed the sustained benefit of clopidogrel over aspirin for long-term maintenance monotherapy (58). While the original HOST-EXAM population consisted predominantly of CCS patients, the consistency of benefit across patient subgroups provides supportive evidence for the potential role of P2Y12 inhibitor monotherapy after DAPT completion, though ACS-specific randomized data remain needed.

Similarly, ULTIMATE-DAPT, which studied ACS patients, showed that switching to ticagrelor monotherapy after 1 month of DAPT (aspirin + ticagrelor) reduced bleeding (1.3% vs. 2.5%, *p* = 0.03) while maintaining comparable MACCE rates (4.1% vs. 4.8%, *p* = 0.62) (5). The GLOBAL LEADERS ACS subgroup analysis and the STOPDAPT-2 ACS trial further support that ticagrelor—but not clopidogrel—monotherapy may provide safe and effective ischemic protection after abbreviated DAPT in ACS patients (59).

Based on these findings, the 2023 ESC guidelines provide a Class IIb recommendation for P2Y12 inhibitor monotherapy after DAPT (Level A evidence). The 2025 ACC guidelines further recommend ticagrelor monotherapy starting 1 month post-PCI in ACS patients at high bleeding risk as a Class I recommendation (Level A evidence) (51).

## 6 Special populations and clinical considerations

### 6.1 Elderly patients

Subgroup analyses of the PLATO trial demonstrated that ticagrelor benefits patients across all age groups. However, a large real-world Chinese study including 18,244 ACS patients aged ≥75 years found that ticagrelor was associated with a significantly higher risk of in-hospital major bleeding compared with clopidogrel, while MACCE rates were similar. For elderly patients with concomitant high bleeding risk (HBR), a short course of potent DAPT followed by de-escalation to clopidogrel may be considered (Class IIb recommendation).

### 6.2 Patients undergoing complex PCI

Complex PCI—characterized by multi-vessel disease or total stent length >60 mm—is generally associated with elevated ischemic risk. Nevertheless, MASTER-DAPT subgroup analysis showed that in patients with both complex PCI and HBR, 1-month DAPT yielded comparable NACE and MACCE rates to longer DAPT durations, while significantly reducing bleeding. This suggests that when bleeding risk outweighs ischemic risk, early de-escalation can be considered even in patients with complex lesions.

### 6.3 Patients requiring oral anticoagulation

Management of ACS patients with atrial fibrillation who require oral anticoagulation (AF-PCI) is of substantial clinical importance. The AUGUSTUS trial demonstrated that apixaban combined with clopidogrel (dual antithrombotic therapy, DAT) significantly reduced bleeding compared with a vitamin K antagonist plus DAPT, without increasing ischemic events, although stent thrombosis showed a trend toward higher incidence (HR 1.59, *p* = 0.04). Current consensus recommends triple therapy (TAT) during the first week post-PCI, followed by DAT (NOAC + P2Y12 inhibitor), preferentially using clopidogrel rather than ticagrelor. A recent review provides comprehensive guidance on the optimal antithrombotic regimen in AF-PCI patients, emphasizing the importance of early aspirin discontinuation (within 1 week) and maintenance of NOAC plus clopidogrel for up to 12 months in most patients (60).

### 6.4 The role of cangrelor in peri-procedural ischemic-hemorrhagic balance

Cangrelor is an intravenous, reversible P2Y12 receptor inhibitor characterized by rapid onset and offset of action, making it particularly suitable for peri-procedural use in patients undergoing PCI who are P2Y12 inhibitor-naïve. Randomized trials (CHAMPION PCI, CHAMPION PLATFORM, CHAMPION PHOENIX) and a patient-level meta-analysis have demonstrated that cangrelor reduces periprocedural ischemic events—including stent thrombosis and periprocedural MI—compared with oral clopidogrel or placebo, albeit with an increase in minor bleeding. Major and life-threatening bleeding rates are similar between cangrelor and control arms. An updated review provides a comprehensive assessment of the ischemic-hemorrhagic balance in patients treated with cangrelor during PCI (61). Current 2023 ESC guidelines assign cangrelor a Class IIb recommendation (Level of Evidence A) for use in P2Y12 inhibitor-naïve ACS patients undergoing PCI, though it has not yet become standard therapy in unselected ACS patients. In the context of de-escalation, cangrelor may be particularly valuable in bridging scenarios where oral P2Y12 inhibitors are withheld peri-procedurally, allowing clinicians to maintain effective platelet inhibition while avoiding excessive bleeding.

### 6.5 Other special populations

Women, low-body-weight patients, and those with end-stage renal disease (CKD stage 5) lack dedicated high-quality RCT data. However, observational studies consistently indicate elevated bleeding risk in these populations. Clinicians should consider these factors when assessing bleeding risk and more actively apply de-escalation strategies in these high-risk groups.

### 6.6 Critical appraisal: study discrepancies and limitations

While the reviewed trials consistently support the net clinical benefit of DAPT de-escalation, several important discrepancies warrant critical appraisal. Notably, the TWILIGHT trial (3-month DAPT followed by ticagrelor monotherapy) demonstrated significant bleeding reduction without ischemic harm in high-risk patients, whereas the STOPDAPT-2 ACS trial raised concerns about ischemic risk with 1-month DAPT followed by clopidogrel monotherapy. These differences likely reflect variations in: (1) the duration of initial DAPT (3 months vs. 1 month); (2) the potency of the P2Y12 inhibitor used for monotherapy (ticagrelor vs. clopidogrel); and (3) baseline patient ischemic risk profiles. The NEO-MINDSET findings further highlight that very early aspirin withdrawal (within days) is not safe, whereas TARGET-FIRST demonstrates that after 1 month of stabilization, aspirin withdrawal can be safe in selected low-risk patients. Collectively, these discrepancies underscore that de-escalation is not a one-size-fits-all concept; rather, the optimal approach depends critically on the timing of de-escalation, the choice of monotherapy agent, and individual patient risk stratification.

Several limitations affect the generalizability of current evidence. Most key trials excluded patients with STEMI (or enrolled them only in small proportions), those with extremely high ischemic-risk lesions (e.g., left main disease, chronic total occlusions), and those with complex anatomical or clinical features. The external validity of East Asian trial data to Western populations—and vice versa—may be limited by the “East Asian paradox” phenomenon (10). Additionally, most studies were open-label, introducing potential bias in outcome assessment. The use of composite endpoints that combine ischemic and bleeding events may obscure differential treatment effects on each component. These limitations should be considered when applying trial findings to individual patients in clinical practice.

## 7 Conclusion

In summary, DAPT after PCI in ACS patients has entered an era of individualized de-escalation. Evidence suggests that after the early high-thrombotic-risk period (typically the first month), timely de-escalation strategies—including switching from potent to milder P2Y12 inhibitors, shortening DAPT to 3–6 months, or maintaining P2Y12 inhibitor monotherapy instead of aspirin—can effectively reduce bleeding without significantly increasing ischemic events, thereby improving net clinical benefit. These approaches are particularly relevant for elderly patients, East Asian populations, and those with multiple bleeding risk factors. Structured risk assessment using PRECISE-DAPT and ARC-HBR criteria is essential for identifying appropriate candidates.

Nonetheless, current de-escalation strategies have notable limitations. Most trials excluded STEMI patients and those with extremely high ischemic-risk lesions. The safety of ultra-short 1-month DAPT in broad ACS populations is well established only for HBR patients, and very early aspirin withdrawal (within days) is not safe based on NEO-MINDSET. While genotype- or platelet-function-guided de-escalation is conceptually appealing, robust evidence from large-scale trials confirming clinical benefit is lacking. High-quality data are also limited for women, low-body-weight patients, and those with end-stage renal disease.

Future research should focus on prospective, China-centric studies that integrate clinical risk scores, imaging assessments, and biomarkers to construct a precise, dynamic risk stratification system. Such efforts will allow long-term validation of various de-escalation strategies and facilitate a true shift from “one-size-fits-all” to personalized antiplatelet therapy in ACS. The upcoming LEGACY trial and ongoing investigations into the optimal timing of aspirin withdrawal will further refine our approach to DAPT de-escalation.

## Data Availability

The original contributions presented in the study are included in the article/supplementary material, further inquiries can be directed to the corresponding author.
